# Functional Somatic Symptoms and Emotion Regulation in Children and Adolescents

**DOI:** 10.32872/cpe.4299

**Published:** 2022-06-30

**Authors:** Stefanie M. Jungmann, Louisa Wagner, Marlene Klein, Aleksandra Kaurin

**Affiliations:** 1 Department of Clinical Psychology, Psychotherapy, and Experimental Psychopathology, Johannes Gutenberg-University Mainz, Mainz, Germany; 2 Department of Clinical Psychology and Psychotherapy, University Witten/Herdecke, Witten, Germany; Philipps-University of Marburg, Marburg, Germany

**Keywords:** adolescents and children, alexithymia, emotion regulation, functional somatic symptoms, parents, transgenerational

## Abstract

**Background:**

Functional Somatic Symptoms (FSS; i.e. symptoms without sufficient organic explanation) often begin in childhood and adolescence and are common to this developmental period. Emotion regulation and parental factors seem to play a relevant role in the development and maintenance of FSS. So far, little systematic research has been conducted in childhood and adolescence on the importance of specific emotion regulation strategies and their links with parental factors.

**Method:**

In two studies, children and adolescents (Study 1/Study 2: N = 46/68; 65%/60% female, Age M = 10.0/13.1) and their parents completed questionnaires on children's FSS and adaptive and maladaptive emotional regulation (in Study 2, additionally parental somatization and child/parental alexithymia).

**Results:**

In both studies, child-reported FSS were negatively associated with children's adaptive emotion regulation (r = -.34/-.31, p < .03; especially acceptance) and positively with children's maladaptive emotion regulation and alexithymia (r = .53/.46, p < .001). Moreover, children’s maladaptive emotion regulation (β = .34, p = .02) explained incremental variance in child-reported FSS beyond children’s age/sex, parental somatization and emotion regulation. In contrast, parental somatization was the only significant predictor (β = .44, p < .001) of parent-reported FSS in children/adolescents.

**Conclusion:**

Our results suggest that particularly rumination and alexithymia and parental somatization are important predictors of FSS in children/adolescents. Overall, the results showed a dependence on the person reporting children's FSS (i.e., method-variance). So, for future studies it is relevant to continue using the multi-informant approach.

## Highlights

- Two Studies found negative associations between child-reported FSS and adaptive emotion regulation.
- We found positive associations between child-reported FSS and maladaptive emotion regulation.
- Parental somatization was the only significant predictor of parent-reported FSS.
- Dependence on the rater stresses the importance of the multi-informant approach.

About 10–25% of children and adolescents suffer from Functional Somatic Symptoms (FSS), i.e. bodily complaints such as abdominal pain or headaches that cannot be sufficiently explained by an underlying physical condition (Berntsson & Köhler, 2001; Rask et al., 2009). These bodily complaints interfere with daily activities and potentially impair academic and psychosocial functioning. Children and adolescents suffering from bodily complaints report frequent absences from school, absent-mindedness, impaired leisure behavior, and lower levels of life quality (Beck, 2008; Hoftun et al., 2011; Malas et al., 2017), FSS represent a key feature of Somatoform Disorders (according to ICD-10; World Health Organization, 1993) or Somatic Symptom Disorders (according to DSM-5; American Psychiatric Association, 2013), (functional) somatic symptoms also co-occur with a variety of other disorders and are thus of transdiagnostic relevance (Aldao et al., 2010; Dufton et al., 2009; Tegethoff et al., 2015).

According to the *perseverative cognition hypothesis* (Brosschot et al., 2006), a preoccupation with stressful events or chronic stress may increase the likelihood to experience bodily symptoms through physiological activation. In adulthood, the importance of affect-regulatory processes to FSS and Somatoform Disorders is well established (Bailer et al., 2017; Schwarz et al., 2017). Previous studies found that negative affect or depression and anxiety disorders are associated with reports of bodily symptoms (Bekhuis et al., 2015; Watson & Pennebaker, 1989; Wessely et al., 1999). Moreover, difficulties in emotion processing, expression, and regulation have been reported to be related to higher levels of FSS (Okur Güney et al., 2019; Schwarz et al., 2017). Adaptive emotion regulation strategies such as reappraisal were negatively and maladaptive strategies such as expressive suppression and alexithymia were positively associated with FSS (Brooks et al., 2017; Erkic et al., 2018). The construct alexithymia describes difficulties in recognizing and describing one's own feelings and is associated with deficits in emotional processing and dysfunctional emotion regulation (Bagby et al., 1994; Luminet et al., 2021).

Less attention has been paid to the relationship between emotional dysregulation and FSS in childhood and adolescence. For instance, in a sample of youth with recurrent abdominal pain (7–18 years), coping strategies including regulating attention or cognitions (“secondary control engagement” such as e.g., acceptance and distraction) were associated with fewer bodily symptoms, and involuntary engagement (including e.g., rumination and intrusive thoughts) with higher levels of bodily symptoms (Thomsen et al., 2002). Regarding emotion-focused (dealing with the emotional experience) and body-focused (dealing with bodily experience, e.g. taking a pill) regulation strategies, children with functional abdominal pain (8–13 years) showed the highest level of body-focused regulation compared to children with no/few and many bodily symptoms, whereas the three groups did not differ in emotion-focused regulation (Rieffe et al., 2007). Gilleland, Suveg, Jacob, and Thomassin (2009) found that child-reported FSS were associated with reduced emotional awareness. Mother-reported FSS in children/adolescents, in addition, was associated with low emotion regulation abilities including low emotion expression, empathy, and self-awareness. While there are hardly any studies on specific emotion regulation strategies in the context of FSS in children and adolescents, some previous studies have focused on emotion awareness and alexithymia. A further study (Jellesma et al., 2006) found that children with many somatic symptoms (highest 30% of a symptom scale) as well as a clinical group of children with functional abdominal pain (8–13 years) reported significantly stronger negative affect and more difficulties in differentiating and communicating feelings compared to children with few somatic symptoms (whereas the first two groups do not differ in that regard). According to a recent systematic review, seven of eight identified studies on self-reported alexithymia showed that children with FSS reported significantly higher levels of alexithymic traits compared to healthy controls (Hadji-Michael et al., 2019).

In terms of a development perspective it is important to consider the development and maintenance of youth FSS in interaction with parental and family factors (Beck, 2008). Models of transgenerational transmissions of psychopathology (Hosman et al., 2009) and particularly those with a focus on pain or emotion regulation (Morris et al., 2007; Stone & Wilson, 2016) suggest that parenting (especially e.g., regarding coping with affect) and children’s emotion regulation might account for the relationship between parental emotional dysregulation/psychopathology and children’s psychological outcomes. Gilleland et al. (2009) found that parental somatization and youth deficits in emotion regulation were significant predictors of mother-reported child somatization and only parental somatization was a significant predictor of father-reported child somatization. In line with the transmission model (Stone & Wilson, 2016), the association between parental pain catastrophizing and adolescent symptom-related impairment was shown to be mediated by the pain catastrophizing of adolescents (Wilson et al., 2014).

So far, there is relatively little research on the relationship between emotion regulation and FSS in the field of clinical child and adolescent psychology. In particular, there is hardly any study on concrete emotion regulation strategies (e.g., reappraisal, suppression, see findings above in adulthood) and very little on the role of parental somatization. Therefore, to narrow this gap in the literature, in two studies we aimed at systematically investigating the relationships between parent and child emotion regulation and parent and child somatization (parental somatization only assessed in Study 2).

Study 1 was designed as a pilot study and investigated relationships between child and parental emotion regulation and children’s FSS. We hypothesized that child and parental reappraisal and acceptance would be negatively associated and rumination and catastrophization would be positively associated with children’s FSS. Based on the transgenerational model (Stone & Wilson, 2016) and previous findings, suggesting that female gender and increasing age in youth are associated with FSS (Lieb et al., 2000; Stone & Wilson, 2016), it was assumed that beyond age and gender and parental emotion regulation, child emotion regulation is a significant predictor of FSS in children and adolescents.

Study 2 aimed at replicating the results of Study 1 in a second sample of youth and their parents (and child and parental alexithymia and parental somatization were assessed). In addition to the hypotheses in Study 1, we expected a positive relationship between child and parental alexithymia and FSS. To expect a specific relationship between emotion regulation and FSS, we also hypothesized that beyond parental somatization, parental and child emotion regulation would explain additional variance in FSS in children and adolescents.

In line with current dimensional-hierarchical approaches to psychopathology (Hierarchical Taxonomy of Psychopathology; HiTOP; Conway et al., 2019) and the preference for the dimensional view especially of somatic symptoms (Jasper et al., 2012), FSS were investigated in the general population.

## Method

### Participants

The participants of Study 1 and 2 were recruited in the general population using flyers posted in schools in German cities. The inclusion criteria were an age of the children and adolescents between 7-14 (Study 1)/8-17 years (Study 2) and a consent of a parent or guardian. There was no drop-out in Study 1. In Study 2 (conducted online), originally *N* = 79 children and parents (each) participated. Due to incorrect completion of the child version by parents and unassigned codes of child and parent, *N* = 11 cases had to be excluded. The socio-demographic data of both samples (*N* = 46/*N* = 68) are shown in Table 1.

**Table 1:** Sociodemographic Characteristics of Children/Adolescents and Their Parents for Study 1 (N = 46) and Study 2 (N = 68)

Sociodemographic variables	Study 1		Study 2	
	*N* (%)	*M* (*SD*)	*N* (%)	*M* (*SD*)
Age				
Children		9.96 (1.58)		13.09 (2.22)
Parents		44.74 (4.63)		44.97 (6.19)
Sex				
Children (female)	30 (65.2%)	–	41 (60.3%)	–
Parents (female)	40 (87.0%)	–	62 (91.2%)	–
Children: Type of school				
Elementary school	29 (63.0%)	–	6 (8.8%)	–
Grammar school	15 (32.6%)	–	25 (36.8%)	–
Secondary school			1 (1.5%)	–
Comprehensive school	–	–	35 (51.5%)	–
Other school type/no statement	2 (4.3%)	–	1 (1.5%)	–
Parents: native language^a^				
German	34 (73.9%)	–	61 (89.7%)	–
Others/no statement	12 (26.1%)		7 (10.3%)	
Parents: Family status				
Married or partnership	34 (73.9%)	–	57 (83.8%)	–
Single/Divorced/Widowed	6 (13.0%)	–	11 (16.2%)	–
No statement	6 (13.0%)	–	–	–
**Parents: Education** (% higher education)	24 (52.2%)	–	29 (42.6%)	–
Parents: Occupation				
Unemployed	1 (2.2%)	–	1 (1.5%)	–
In training	–	–	1 (1.5%)	–
Employee/civil servant	30 (65.2%)	–	53 (77.9%)	–
Self-employed	2 (4.3%)	–	5 (7.4%)	–
Housewife/-husband	10 (21.7%)	–	7 (10.3%)	–
Retired	–		1 (1.5%)	
No statement	3 (6.5%)	–	–	–

^a^Study 2 also asked for nationality: 95.6% German or dual citizenship including German (e.g. Czech, Romanian, Croatian), 1.5% American, 1.5% Bulgarian and 1.5% Czech.

### Procedure

The data collection of *Study 1* was part of a larger project on the behavioral assessment of psychopathology in children and adolescents from the general population (in 2017-2018). The study took place on a single date in a research laboratory of the Psychological Institute. The study design required the accompaniment of one parent, which one was not pre-determined by the study, but according to the time capacity of the parents (in 87% of the cases the mother as accompaniment). As compensation, the children received a small game (e.g., board game) and the parents 10€ per hour. *Study 2* was an online study about body awareness and dealing with feelings (over 8 weeks in 2019). In both studies, parents and children/adolescents gave informed consent prior to participation. As compensation for their participation, children and parents were given the opportunity to take part in a lottery for 5 x 15 € gift vouchers. The study protocols of both studies were approved by the institutional review board of the Psychological Institute.

### Measures

#### Children and Adolescents

The *Screening for Somatoform Disorders in Children and Adolescents* (SOMS-CA; Winter et al., 2018) is a validated self-reported measure for assessing FSS. The *SOMS-CA* was used in *Study 1 and 2*. Participants report on 33 somatic symptoms (pain, gastrointestinal, cardiorespiratory, and pseudo-neurological symptoms) that have occurred in the last 6 months and for which the doctor has not found a clear medical explanation. A total number of complaints is calculated from the sum of the 33 bodily symptoms. This score showed high internal consistencies in both studies (Cronbach’s α = .91/.84). Additionally, the *SOMS-CA* assesses further characteristics of FSS, such as illness-related behavior (e.g., doctor visits) and functional impairments. An additional score can be calculated including these factors, whereby an earlier study (Jungmann & Witthöft, 2020) showed that this score can be ambiguous due to jump rules, so this study focused on the above-mentioned total number score of FSS.

The *Questionnaire to assess Emotion Regulation in Children and Youths* (Fragebogen zur Erhebung der Emotionsregulation bei Kindern und Jugendlichen, FEEL-KJ; Abler & Kessler, 2009) is a validated 90-item self-report measure for assessing different strategies of emotion regulation when children and adolescents feel sad, anxious, and angry. Emotion regulation can be divided into the superordinate scales “adaptive” (e.g., acceptance and reappraisal), “maladaptive” (e.g., rumination), and “other strategies” (e.g., social support). A five-point Likert scale (1 = *almost never* to 5 = *almost always*) is used to indicate the degree of agreement with each statement. The *FEEL-KJ* showed acceptable to high reliability as well as construct and external validity (Cracco et al., 2015). Both studies focused on “adaptive” (α = .95/.85), “maladaptive emotion regulation” (α = .91/.77), and the individual strategies of “acceptance” (α = .75/.56), “reappraisal” (α = .76/.52), and “rumination” (α = .77/.46). Based on previous studies on the links between depression, emotion regulation, and bodily complaints (Allen et al., 2011), *Study 2* focused only on strategies in response to sadness (each strategy with two items).

Additionally, *Study 2* used the *Alexithymia Questionnaire for Children* (AQC; Rieffe et al., 2006), a 20-item self-report questionnaire for assessing alexithymia in children/adolescents. The items (e.g., "I don't know what's going on inside me.") are rated on a three-point Likert scale (0 = *not true* to 2 = *often true*). The internal consistency was α = .82.

#### Parents

The *Screening for Somatoform Disorders in children and adolescents for parents* (SOMS-P; Voß, 2013) measures the severity of children’s FSS from the parents’ perspective. The structure and scoring are analogous to the SOMS-CA (α = .82/.81).

The *Cognitive Emotion Regulation Questionnaire* (CERQ; Loch et al., 2011) assesses cognitive emotion regulation strategies used in the context of negative experiences or life events. The frequency of using the different strategies is measured with a five-point Likert scale (1 = *almost never* to 5 = *almost always*). Based on our hypotheses, both studies focused on the superordinate scales “adaptive” (α = .93/.80) and “maladaptive emotion regulation” (α = .81/.72) and on the individual strategies “acceptance” (α = .84/.82), “reappraisal” (α = .85/.63), “rumination” (α = .66/.53), and “catastrophizing” (α = .65/.73). Following Garnefski and Kraaij (2006), *Study 2* used a shortened version with two items per strategy.

Additionally, *Study 2* included the *Toronto Alexithymia Scale* (TAS; Bagby et al., 1994; Popp et al., 2008) and the *Brief Symptom Inventory* (BSI; Franke, 2000). The *TAS-20* is a 20-item self-report measure for assessing alexithymia in adults and comprises a five-point Likert scale from 1 = *not at all true* to 5 = *completely true* (α = .85). The *BSI* is a 53-item screening questionnaire assessing various psychopathological characteristics within the last 7 days in adulthood. The items are rated on a scale from 0 = *not at all* to 4 = *very strong*. Based on our hypotheses, we have focused on the subscale of somatization (7 items, α = .62).

### Statistical Analyses

Statistical analyses were carried out with SPSS 23.0. For Study 2, an a priori power analysis using G*Power with ρ H1 = .4, an alpha error = .05, and a statistical power = .90 for bivariate correlations resulted in a minimum sample size of *N* = 61 (not for Study 2 due to a pilot study as a part of a larger project). In the online study (Study 2), the survey response was set so that no questions could be omitted. In Study 1, the pairwise deletion method was used for individual missing items. To test the relationships, we first calculated Pearson correlation coefficients (most variables were approximately normally distributed). To examine the incremental variance explained by parental/child emotion regulation, multiple hierarchical regression models were computed for the dependent variable FSS (once for child-reported/parent-reported FSS). For regression analyses, first multicollinearity was checked (correlations of the predictors were each below *r* = .70; Tabachnick & Fidell, 1996). In *Study 1*, sex and age were controlled for in Step 1, parental adaptive and maladaptive emotion regulation were entered in Step 2 (CERQ), and child emotion regulation in Step 3 (FEEL-KJ) (order in line with the transgenerational model; Stone & Wilson, 2016). In *Study 2*, parental somatization was added in Step 2 and parental/child emotion regulation in Steps 3 and 4 (CERQ, FEEL-KJ) (order in line with Gilleland et al., 2009).

## Results

### Participant Characteristics Regarding FSS and Emotion Regulation

In *Study 1*, boys and girls did not differ significantly in FSS and emotion regulation (*p* ≥ .23, *d* ≤ 0.40). With regard to age of the children, significant positive correlations were found with children’s maladaptive emotion regulation (*r* = .38, *p* = .011) and children’s rumination (*r* = .33, *p* = .030).

In *Study 2*, girls showed higher scores compared to boys in reporting maladaptive emotion regulation, *t*(66) = -2.97, *p* = .004, *d* = 0.73, and rumination, *t*(66) = -3.18, *p* = .002, *d* = 0.78. Children’s age correlated positively with the child-reported gastrointestinal symptoms (*r* = .30, *p* = .015) and maladaptive emotion regulation (*r* = .32, *p* = .008). Appendix A (see Supplementary Materials) shows the participant characteristics of *Study 1* and *2*.

### Relationships Between FSS and Emotion Regulation

#### Study 1

As expected, in *Study 1* we found a negative correlation between children’s adaptive emotion regulation and child-reported FSS (*r* = -.34, *p* = .026). At the level of individual strategies, there were no significant associations with reappraisal (*r* < .01, *p* > .99) and acceptance (*r* = -.26, *p* = .096). As hypothesized, there were significant positive correlations between children’s maladaptive emotion regulation (*r* = .53, *p* ≤ .001) and rumination (*r* = .41, *p* = .001) with child-reported FSS. No significant correlations were found between children’s emotion regulation and parent-reported FSS in children/adolescents (*r* ≤ |.20|, *p* ≥ .198). Concerning parental emotion regulation, as expected, parental rumination was significantly positively associated with child-reported FSS (*r* = .34, *p* = .028) and parental maladaptive emotion regulation (*r* = .37, *p* = .011) was well as parental rumination (*r* = .37, *p* = .011) were significantly positively correlated with parent-reported FSS. Appendix B (see Supplementary Materials) describes the correlations between child and parental somatization and child and parental emotion regulation.

#### Study 2

As in Study 1, *Study 2* found a significant negative correlation between children’s adaptive emotion regulation and child-reported FSS (*r* = -.31, *p* = .011). At the level of individual strategies, there was a significant negative association between children’s acceptance and child-reported FSS (*r* = -.36, *p* = .003). Children’s maladaptive emotion regulation (*r* = .46, *p* ≤ .001) and alexithymia (*r* = .39, *p* = .001) were significantly positively correlated with child-reported FSS. Additionally, children’s acceptance was significantly negatively correlated with parent-reported FSS (*r* = -.25, *p* = .047). Regarding parental emotion regulation, parental acceptance showed a significant negative correlation with child-reported FSS (*r* = -.30, *p* = .018). In addition, parental maladaptive emotion regulation (*r* = .29, *p* = .018) and alexithymia (*r* = .29, *p* = .018) were positively correlated with parental somatization (see Appendix B in the Supplementary Materials).

### Regression Analyses for Predicting FSS in Children and Adolescents

#### Study 1

In *Study 1*, children’s emotional regulation (∆*R*^2^ = .34, *p* = .001) explained variance in child-reported FSS over and above children’s age/gender and parental emotional regulation (Appendix C, Supplementary Materials). As the correlations showed, both child adaptive (β = -.30, *p* = .040) and maladaptive emotion regulation (β = .50, *p* = .002) were significant predictors of child-reported FSS. To investigate specific regulation strategies, this multiple hierarchical regression was repeated by using the specific hypothesized emotion regulation strategies (acceptance, reappraisal, and rumination) instead of general adaptive and maladaptive emotion regulation. Child acceptance (β = -.36, *p* = .028) was found to be a negative predictor and rumination a positive predictor (β = .46, *p* = .007) of child-reported FSS.

The same analyses were carried out for the dependent variable parent-reported FSS (Appendix D, Supplementary Materials). In this model, children’s emotional regulation showed no incremental explanation for variance in parent-reported FSS (∆*R*^2^ = .01, *p* = .892) in addition to age/gender, and parental emotional regulation. Parental emotion regulation provided a significant explanation of variance in parent-reported FSS (∆*R*^2^ = .22, *p* = .010), which can be attributed to maladaptive emotion regulation as a significant predictor (β = .37, *p* = .025). Including individual strategies (catastrophization and rumination), parental rumination (β = .48, *p* = .017) was found to demarcate a significant predictor of parent-reported FSS.

#### Study 2

As in Study 1, in *Study 2* children’s emotion regulation explained incremental variance in child-reported FSS (∆*R*^2^ = .14, *p* = .009) beyond age/sex, parental somatization, and parental emotion regulation (Appendix C, Supplementary Materials). Maladaptive emotion regulation (β = .34, *p* = .020) was shown to be a significant predictor of child-reported FSS, whereby the assumed individual strategy rumination did not prove to be a significant predictor (β = .04, *p* = .777)

This analysis was repeated for the dependent variable parent-reported FSS in children/adolescents (Appendix D, Supplementary Materials). As in Study 1, in *Study 2* children’s emotion regulation did not explain significant incremental variance in parent-reported FSS (∆*R*^2^ = .014, *p* = .594) beyond age/sex, parental somatization, and parental emotion regulation. In this model for predicting parent-reported FSS, parental somatization was the only significant predictor (β = .44, *p* < .001).

## Discussion

Two studies were conducted to investigate the relationships between child and parental emotional regulation and child and parental somatization. Based on previous research and the transgenerational model for the development of FSS in children/adolescents (Gilleland et al., 2009; Stone & Wilson, 2016), we hypothesized that children’s emotion regulation should explain additional variance in children’s FSS beyond parental somatization and emotional regulation. We tested our hypotheses in a pilot sample, and then replicated the findings in an independent sample.

To evaluate the levels of FSS in our studies, we have set them in relation with a previous study among children/adolescents in the general population (Jungmann & Witthöft, 2020). Compared to the study by Jungmann and Witthöft (2020), in the present studies the total number of child-reported FSS was higher (*d* = .31/.66). Regarding socio-demographic data, the children in our two studies were on average younger (*M* = 10.0/13.1 vs. *M* = 14.2 in Jungmann & Witthöft, 2020), the gender distribution was comparable (59 – 65% female). There are inconsistent findings on the relationship between FSS and age, Lieb et al. (2000), for example, describe a steep increase between the ages of 8 and 12, other studies found no relationship between age and FSS in children and adolescents (Cerutti et al., 2017; Dhossche et al., 2001). Also, only Study 2, but not Study 1, found a significant correlation between age and gastrointestinal symptoms. Presumably, additional factors or an interaction of factors can better explain the level of FSS. Since Jungmann and Witthöft's (2020) study used the same measuring instrument to record FSS, situational factors (e.g., holidays), the type of survey (laboratory/at home), and/or parent-child interactions (e.g., parents’ reactions to child’s symptoms) would be conceivable. The latter point could also be in line with the transgenerational model (Stone & Wilson, 2016), which assumes that the parental influence on the perception and expression of body symptoms is greater in younger children.

In accordance with the hypothesis and consistently in both studies, significant negative correlations were found between children’s adaptive emotion regulation and child-reported FSS. This is also compatible with previous studies on bodily complaints in children and adolescents, whereby the present studies have examined more specifically adaptive emotion regulation in comparison with coping processes (Thomsen et al., 2002) and emotion regulation abilities such as empathy and self-awareness (Gilleland et al., 2009). As assumed, Study 2 also found a negative association between acceptance and child-reported FSS, in accordance with the study by Thomsen et al. (Thomsen et al., 2002) in which acceptance represented a kind of secondary control engagement. Possible reasons why Study 1 missed the significance level for this association (*p* = .096) could be the smaller sample, but also, for example, the younger age. Possibly, younger children may use this strategy less or have less understanding of what it meant (e.g., "I accept what makes me angry."). In contrast to the study by Erkic et al. (2018), which showed a reduced level of the reappraisal strategy in adults with SSD, no significant correlations between children’s reappraisal and child-reported FSS were found in both studies. On the one hand, this strategy could be less developed in childhood, which is also shown by the fact that the mean scores for reappraisal were lower than those for acceptance; on the other hand, this correlation could also only become apparent in the pathological manifestation of SSD.

As expected and consistent in both studies, positive associations between childhood maladaptive emotional regulation and child-reported FSS were also found. Only Study 1 showed a significant positive correlation with rumination. In Study 2, the subscale rumination showed a low internal consistency (α = .46), which could possibly be due to the fact that rumination in terms of the shortened version of the FEEL-KJ was recorded with only two items. In addition, our study also confirmed the positive correlation between alexithymia and child-reported FSS (Hadji-Michael et al., 2019).

In comparison to the relationships between the child reports, only a significant association was found between child acceptance and parent-reported FSS. This association might indicate that when children show higher acceptance, parents perceive or report less body symptoms of their children. Moreover, in line with previous studies (De Los Reyes et al., 2015), this finding also suggests that the child's and parent's judgements can differ more significantly in the case of personal experiences and internalizing symptoms, and consequently it is relevant (even if the children are younger) to question the children themselves.

Explaining child-reported FSS, children’s emotional regulation consistently showed the highest variance explanation in both studies (14–34%) and explained additional variance in addition to age/gender and parental emotional regulation. Especially maladaptive emotion regulation was found to be a significant predictor. This suggests the importance of children’s emotional regulation for children’s FSS and confirms findings in adults (Erkic et al., 2018). Like first approaches in adulthood (Kleinstäuber et al., 2019), the promotion of adaptive emotion regulation/reduction of dysfunctional emotion regulation could be a promising approach for the psychotherapeutic treatment of FSS in childhood and adolescence. For variance explanation of parent-reported FSS, parental emotion regulation (22%), especially parental maladaptive emotion regulation, showed a significant incremental variance explanation in Study 1, but when parental somatization was also included in Study 2, it was the only significant predictor. Thomsen et al. (2002) also found only parental somatization as a significant predictor of father-reported physical complaints in children. This result could indicate that the estimation/perception of childhood FSS depends on the parents' own experience of physical complaints, which should also be taken into account, for example, when exploring/treating FSS in children. The findings could also be consistent with current interoceptive predictive coding models of symptom perception (e.g., Van den Bergh et al., 2017) which assume that the perception and evaluation of body symptoms is influenced by previous experience. This could be the case not only for the perception of one's own body symptoms, but also for those of children.

Some limitations should be mentioned. The samples of both studies are rather small (especially Study 1, see also power analysis) and not representative in terms of socio-demographic data (e.g., parents’ high education, 80–90% mothers). Our cross-sectional design does not allow us to draw any causal conclusions; longitudinal studies would also be of interest, e.g., to examine the temporal course of deficits in the emotion regulation of FSS and the transgenerational model more closely. The survey conditions (Study 1/2: laboratory/Online), samples (age), and, in some cases, the measuring instruments differ between Study 1 and 2. We cannot exclude the influence of these factors on our results. For example, the partly found divergences of Study 1 and Study 2 might have resulted from different survey conditions. In Study 2, the shortened version of the FEEL-KJ found partially low internal consistencies of the individual strategies, which should be examined in further studies. In this context, it should also be mentioned that some questionnaires for children are not validated in German or for an age below 10/11 years. Validation studies are needed here in the future. This could contribute to biases (e.g., too low scores) because the items are still too difficult for younger children (8-10 years).

### Conclusion

In summary, our studies indicate that in childhood and adolescence, emotion regulation is related to FSS. Thus, the promotion of functional emotion regulation/reduction of maladaptive emotion regulation likely represents a promising complementary approach for the treatment of FSS in children and adolescents. In predicting parent-reported FSS, parental somatization was the only significant predictor. This finding highlights the dependence on the perspective and previous experience with body symptoms of the person making the assessment. Therefore, the consideration of parental factors is also relevant in the treatment of FSS in children. Furthermore, it shows the importance of multidimensional approaches, whereby in addition to a multi-informant approach the inclusion of experimental procedures could present a key source of information in future studies (e.g., promoting adaptive emotion regulation in children with FSS).

## Supplementary Materials

The Supplementary Materials contain the following items (for access see Index of Supplementary Materials below).

- **Appendix A.** Participant characteristics regarding functional somatic symptoms (FSS) and emotion regulation.
- **Appendix B.** Pearson correlations between FSS and emotion regulation.
- **Appendix C.** Multiple hierarchical regression analyses for predicting *child-reported FSS* in children and adolescents.
- **Appendix D.** Multiple hierarchical regression analyses for predicting *parent-reported FSS* in children and adolescents.

10.23668/psycharchives.6976 Supplement 1 Supplementary materials to "Functional somatic symptoms and emotion regulation in children and adolescents"

JungmannS. M.
WagnerL.
KleinM.
KaurinA.
 (2022). Supplementary materials to "Functional somatic symptoms and emotion regulation in children and adolescents"
[Additional information]. PsychOpen. 10.23668/psycharchives.6976
PMC966741936397947
