# Supplementary material for: Functional Somatic Symptoms and Emotion Regulation in Children and Adolescents
Source: Clin Psychol Eur. 2022 Jun 30;4(2):e4299. doi: 10.32872/cpe.4299 (PMC9667419; doi:10.32872/cpe.4299)
Supplement: Supplement 1 [file cpe-04-4299-s01.pdf]

Functional somatic symptoms and emotion regulation in children and adolescents
Stefanie M. Jungmann, Louisa Wagner, Marlene Klein, Aleksandra Kaurin
Clinical Psychology in Europe
https://doi.org/10.32872/cpe.4299

Appendix A

Participant characteristics regarding functional somatic symptoms (FSS) and emotion regulation in *Study 1* (*N* = 46) and *Study 2* (*N* = 68).

	Study 1			*Study 2*		
**Somatic symptoms**	*M*	*SD*	*Range*	*M*	*SD*	*Range*
Child-reported FSS (SOMS-CA; 0–33*)	5.31	5.91	0-20	6.63	4.84	0-19
Child-reported pain (SOMS-CA; 0–10)	2.02	1.96	0-8	2.58	1.84	0-9
Child-reported gastrointestinal symptoms (SOMS-CA; 0–5)	0.69	1.20	0-4	1.23	1.19	0-4
Child-reported cardiorespiratory symptoms (SOMS-CA; 0–5)	1.40	1.67	0-5	1.46	1.30	0-4
Child-reported pseudo-neurological symptoms (SOMS-CA; 0–12)	1.10	1.90	0-8	1.53	1.64	0-8
Parent-reported FSS (SOMS-P; 0–33*)	2.57	3.26	0-14	3.58	3.52	0-22
Parent-reported pain (SOMS-P; 0–10)	1.43	1.87	0-7	1.80	1.56	0-7
Parent-reported gastrointestinal symptoms (SOMS-P; 0–5)	0.35	0.85	0-4	0.76	1.07	0-4
Parent-reported cardiorespiratory symptoms (SOMS-P; 0–5)	0.59	0.98	0-4	0.70	0.98	0-5
Parent-reported pseudo-neurological symptoms (SOMS-P; 0–12)	0.20	0.54	0-3	0.30	1.07	0-8
Parental somatization (BSI; 0–4)	-	-		0.36	0.37	
**Child emotion regulation**						
Adaptive emotion regulation (FEEL-KJ; 1–5)	2.96	0.67		2.97	0.67	
Acceptance (FEEL-KJ; 1–5)	2.86	0.85		3.09	0.97	
Reappraisal (FEEL-KJ; 1–5)	2.43	0.81		2.49	0.87	
Maladaptive emotion regulation (FEEL-KJ; 1–5)	2.34	0.61		2.91	0.68	

Rumination (FEEL-KJ; 1–5)	2.99	0.92	3.40	0.96
Alexithymia (AQC; 0–2)	-	-	0.82	0.33
**Parental emotion regulation**				
Adaptive emotion regulation (CERQ; 1–5)	2.91	0.73	3.11	0.63
Acceptance (CERQ; 1–5)	2.91	0.98	3.27	0.95
Reappraisal (CERQ; 1–5)	3.11	0.98	3.15	1.01
Maladaptive emotion regulation (CERQ; 1–5)	2.06	0.50	2.35	0.58
Rumination (CERQ; 1–5)	2.42	0.80	2.99	0.98
Catastrophization (CERQ; 1–5)	1.65	0.65	2.34	0.84
Alexithymia (TAS-20; 1–5)	-	-	1.91	0.49

SOMS-CA = Screening for Somatoform Disorders in children and adolescents; SOMS-P = Screening for Somatoform Disorders parent version; BSI = Brief Symptom Inventory; FEEL-KJ = Questionnaire to assess emotion regulation in children and adolescents (Fragebogen zur Erhebung der Emotionsregulation bei Kindern und Jugendlichen); AQC = Alexithymia Questionnaire for children; CERQ = Cognitive Emotion Regulation Questionnaire; TAS-20 = Toronto Alexithymia Scale. [*]Item no. 33 “other complaint” only in the total value. The maximum possible range of answers is indicated in brackets behind the questionnaire or subscale.

Appendix B

Pearson correlations between FSS and emotion regulation in *Study 1* (*N* = 46)/*Study 2* (*N* = 68)

	1	2	3	4	5	6	7	8	9	10	11	12	13	14	15	16
1. Child FSS (SOMS-CA)	1															
2. Child FSS (SOMS-P)	.27/ .51***	1														
3. Parental somatization (BSI)	-/ .17	-/ .44***	1													
4. Child adaptive ER (FEEL-KJ)	-.34*/ -.31*	-.12/ -.22	-/ -.17	1												
5. Child reappraisal (FEEL-KJ)	<.01/ -.20	-.04/ -.19	-/ -.14	.45**/ .65**	1											
6. Child acceptance (FEEL-KJ)	-.26/ -.36**	-.20/ -.25*	-/ -.11	.79***/ .64***	.29/ .43***	1										
7. Child maladaptive ER (FEEL-KJ)	.53***/ .46***	.08/ .16	-/ .22	-.13/ -.32**	.35*/ -.17	-.02/ -.41***	1									
8. Child rumination (FEEL-KJ)	.41**/ .17	.10/ .13	-/ .25*	.03/ -.13	.12/ -.12	.16/ -.26*	.81***/ .69***	1								
9. Child alexithymia (AQC)	-/ .39***	-/ .04	-/ -.14	-/ -.20	-/ -.15	-/ -.26*	-/ .40***	-/ .09	1							
10. Parent adaptive ER (CERQ)	.02/ -.14	.27/ -.17	-/ -.16	-.24/ .20	-.25 .19	-.22/ .13	-.06/ -.28*	<.01/ -.18	-/ -.08	1						
11. Parent reappraisal (CERQ)	-.12/ .06	.24/ -.01	-/ -.07	-.09/ .17	-.17/ .20	-.05/ .16	-.26/ -.24	-.14/ -.14	-/ <.01	.85***/ .78***	1					
12. Parent acceptance (CERQ)	.14/ -.30*	.22/ -.19	-/ -.05	-.17/ .24	-.22/ .19	-.12/ .22	-.01/ -.23	-.02/ -.04	-/ -.02	.80***/ .51***	.60***/ .27*	1				
13. Parent maladaptive ER (CERQ)	.22/ .15	.37*/ .21	-/ .29*	.03/ -.08	.05/ <.01	-.01/ -.14	.18/ .20	.18/ .37**	-/ .18	.05/ .03	.04/ .17	.05/ .12	1			

14. Parent catastrophization (CERQ)	.02/ .13	.21/ .23	-/ .19	.03/ -.11	-.05/ -.05	.06/ -.11	.03/ .19	<.01/ .36***	-/ .17	.16/ -.25*	.15/ -.04	.29*/ -.01	.78***/ .76***	1		
15. Parent rumination (CERQ)	.34*/ .14	.37**/ .07	-/ .19	-.04/ -.06	.12/ <.01	-.08/ -.11	.34*/ .13	.31*/ .34**	-/ .12	-.03/ .16	-.10/ .26*	-.09/ .10	.81***/ .77***	.49***/ .39***	1	
16. Parent Alexithymia (TAS-20)	-/ -.01	-/ .08	-/ .29*	-/ -.01	-/ .02	-/ -.06	-/ .13	-/ .15	-/ .17	-/ -.17	-/ -.15	-/ .16	-/ .25*	-/ .35**	-/ -.03	1

SOMS-CA = Screening for Somatoform Disorders in children and adolescents; SOMS-P = Screening for Somatoform Disorders parent version; BSI = Brief Symptom Inventory; FEEL-KJ = Questionnaire to assess emotion regulation in children and adolescents (Fragebogen zur Erhebung der Emotionsregulation bei Kindern und Jugendlichen; in comparison to Study 1, Study 2 assessed emotion regulation only in response to sadness - not to fear and anger); CERQ = Cognitive Emotion Regulation Questionnaire; AQC = Alexithymia Questionnaire for children; TAS-20 = Toronto Alexithymia Scale. *** $p \leq .001$, ** $p \leq .01$, * $p \leq .05$

Appendix C

Multiple hierarchical regression analyses for predicting *child-reported FSS* in children and adolescents in *Study 1* (N = 46) and *Study 2* (N = 68).

**Study 1**			**Study 2**		
	Coeff.	*p*		Coeff.	*p*
**Step 1**					
age	.06	.72	age	.20	.12
gender	.04	.83	gender	.19	.13
R^2/R^2_{adj}	.01/-.05	.88	R^2/R^2_{adj}	.09/.06	.06
ΔR^2	.01	.88		.09	.06
**Step 2**					
age	.07	.70	age	.18	.16
gender	-.02	.91	gender	.18	.17
			somatization (P)	.11	.38
adaptive (P)	.02	.93			
maladaptive (P)	.21	.24			
R^2/R^2_{adj}	.05/-.06	.78	R^2/R^2_{adj}	.10/.05	.10
ΔR^2	.04	.48	ΔR^2	.01	.38
**Step 3**					
age	-.14	.38	age	.19	.14
gender	-.02	.88	gender	.16	.23
			somatization (P)	.06	.65
adaptive (P)	-.07	.66	adaptive (P)	-.12	.35
maladaptive (P)	.17	.26	maladaptive (P)	.09	.49
adaptive (C)	**-.30**	**.04**			
maladaptive (C)	**.50**	**<.01**			
R^2/R^2_{adj}	**.38/.28**	**<.01**	R^2/R^2_{adj}	.12/.04	.19
ΔR^2	**.34**	**<.01**	ΔR^2	.02	.53
**Step 4**					
			age	.10	.43
			gender	.06	.65
			somatization (P)	.01	.97
			adaptive (P)	-.01	.94
			maladaptive (P)	.05	.71
			adaptive (C)	-.17	.20
			maladaptive (C)	**.34**	**.02**
			R^2/R^2_{adj}	**.26/.16**	**.02**
			ΔR^2	**.14**	**<.01**

FSS = Functional Somatic Symptoms; Adaptive = adaptive emotion regulation; Maladaptive = maladaptive emotion regulation; P = parent, C = child; significant coefficients are bolded.

Appendix D

Multiple hierarchical regression analyses for predicting *parent-reported FSS* in children and adolescents in *Study 1* ($N = 46$) and *Study 2* ($N = 68$).

**Study 1**			**Study 2**		
	Coeff.	*p*		Coeff.	*p*
**Step 1**					
age	.05	.78	age	.02	.86
gender	.00	.99	gender	.12	.34
R^2/R^2_{adj}	.00/-.05	.96	R^2/R^2_{adj}	.02/-.01	.58
ΔR^2	.00	.96		.02	.58
**Step 2**					
age	.08	.62	age	-.06	.64
gender	-.05	.77	gender	.09	.47
			somatization (P)	**.44**	**<.01**
adaptive (P)	.28	.08			
maladaptive (P)	**.37**	**.02**			
R^2/R^2_{adj}	**.22/.14**	**.05**	R^2/R^2_{adj}	**.20/.16**	**<.01**
ΔR^2	**.22**	**.01**	ΔR^2	**.18**	**<.01**
**Step 3**					
age	.08	.63	age	-.05	.70
gender	-.06	.74	gender	.06	.60
			somatization (P)	**.40**	**<.01**
adaptive (P)	.26	.13	adaptive (P)	-.10	.38
maladaptive (P)	**.37**	**.03**	maladaptive (P)	.08	.53
adaptive (C)	-.07	.64			
maladaptive (C)	-.02	.90			
R^2/R^2_{adj}	.23/.10	.15	R^2/R^2_{adj}	**.21/.15**	**.01**
ΔR^2	.01	.89	ΔR^2	.01	.58
**Step 4**					
			age	-.04	.77
			gender	.05	.69
			somatization (P)	**.38**	**<.01**
			adaptive (P)	-.08	.50
			maladaptive (P)	.08	.54
			adaptive (C)	-.13	.32
			maladaptive (C)	-.01	.96
			R^2/R^2_{adj}	**.23/.13**	**.03**
			ΔR^2	.01	.59

FSS = Functional Somatic Symptoms; Adaptive = adaptive emotion regulation; Maladaptive = maladaptive emotion regulation; P = parent, C = child; significant coefficients are bolded.
